# Preparation and characterization of silk fibroin as a biomaterial with potential for drug delivery

**DOI:** 10.1186/1479-5876-10-117

**Published:** 2012-06-07

**Authors:** Hao Zhang, Ling-ling Li, Fang-yin Dai, Hao-hao Zhang, Bing Ni, Wei Zhou, Xia Yang, Yu-zhang Wu

**Affiliations:** 1 Institute of Immunology Third Military Medical University, Chongqing, 400038, Peoples Republic of China; 2 Biochemistry engineering department, Chongqing Industry & Trade Polytechnic, Chongqing, 408000, Peoples Republic of China; 3 State Key Laboratory of Silkworm Genome Biology, Southwest University, Chongqing, 400715, Peoples Republic of China

**Keywords:** Silk fibroin, Calcium-alcohol solutions, Crystalline structure, Drug delivery, Biomaterial

## Abstract

**Background:**

Degummed silk fibroin from *Bombyx mori* (silkworm) has potential carrier capabilities for drug delivery in humans; however, the processing methods have yet to be comparatively analyzed to determine the differential effects on the silk protein properties, including crystalline structure and activity.

**Methods:**

In this study, we treated degummed silk with four kinds of calcium-alcohol solutions, and performed secondary structure measurements and enzyme activity test to distinguish the differences between the regenerated fibroins and degummed silk fibroin.

**Results:**

Gel electrophoresis analysis revealed that Ca(NO_3_)_2_-methanol, Ca(NO_3_)_2_-ethanol, or CaCl_2_-methanol treatments produced more lower molecular weights of silk fibroin than CaCl_2_-ethanol. X-ray diffraction and Fourier-transform infrared spectroscopy showed that CaCl_2_-ethanol produced a crystalline structure with more silk I (α-form, type II β-turn), while the other treatments produced more silk II (β-form, anti-parallel β-pleated sheet). Solid-State ^13^C cross polarization and magic angle spinning-nuclear magnetic resonance measurements suggested that regenerated fibroins from CaCl_2_-ethanol were nearly identical to degummed silk fibroin, while the other treatments produced fibroins with significantly different chemical shifts. Finally, enzyme activity test indicated that silk fibroins from CaCl_2_-ethanol had higher activity when linked to a known chemotherapeutic drug, L-asparaginase, than the fibroins from other treatments.

**Conclusions:**

Collectively, these results suggest that the CaCl_2_-ethanol processing method produces silk fibroin with biomaterial properties that are appropriate for drug delivery.

## Background

Silk fibers produced by silkworms are widely used in our daily life. While they have occupied an important niche in the textile industry for thousands of years, their potential as biomaterials has been recognized and developed only over the past decade [1]. Being non-toxic, non-immunogenic, and biocompatible with a broad range of animal species has allowed for the adherent properties of silk fibroin and silk-like proteins to be exploited for biomedical purposes. To date, silk fibroins have mainly been applied to wound healing, successfully performing as man-made blood-vessels [2], surgical sutures [3], and repair materials [4]. New processing strategies for silk fibers and proteins have expanded the biomedical utility of these molecules. For example, the gel spun silk-based matrix derived from silk fibroin was successfully applied for bladder augmentation in a murine model [5]. More recently, scientists determined that the cocoons from *Bombyx mori* harbor antioxidant and hypolipidemic properties and that the crude silk extracts have bioactivity against hypercholesterolemia and atherosclerosis [6].

Recently, the regenerated silk fibroin has been proved as an attractive candidate of a carrier for drug or therapeutic proteins delivery and is the focus of much ongoing research. Attachment of bioactive molecules or therapeutic proteins to silk fibroin has many benefits to enhance the properties of bioactive molecules in solution for delivery both *in vitro* and *in vivo*, including the therapeutic efficacy in the body, thermal stability, storage stability, and lengthens the circulatory half-life and decreases immunogenicity and antigenicity [3]. For instance, bioconjugations of insulin, glucose oxidase, L-asparaginase (L-ASNase), lipase and phenylalanine ammonia-lyase with the regenerated silk fibroin greatly improved their biological stability, reduced the immunogenicity and toxicity of the drug [7-11]. Moreover, The SELP (silk-elastinlike protein polymer)-controlled gene delivery approach could potentially improve activity of adenoviral-mediated gene therapy of head and neck cancer and limit viral spread to normal organs at the same time [12].

It has been known that the properties of silk-matrix are controlled by a combination of the chemistry and the spinning process, which directly affect the activity and stability of the enzymes attached. Spinning conditions, such as temperature, drawing rate, time, and specific type of silkworm, can modulate biomaterial features. In addition, chemistry, such as ion concentration, type of ion, and solution pH, can also affect the mechanical properties of silk fibroins [1]. In previous studies, degummed fibroin has generally been treated with aqueous solutions of hexafluoro-isopropanol (HFIP) [13], methanol [8], CaCl_2_-ethanol [7,9], or Ca(NO_3_)_2_-methanol [14]. Lu et al. has reported glucose oxidase attached to the regenerated silk fibroin film without treated with methanol remain more activity but lower stability than that treated with methanol [8]. After cross-linking L-ASNase with regenerated silk fibroin prepared with concentrated CaCl_2_ mixture solution with ethanol and water (1:2:8, mol), the immunogenicity and toxicity of the drug significantly reduced, and its circulatory half-life lengthened *in vitro*[9].

However, these studies have used only one treatment per experiment and, up to now, the systematic comparative analysis to distinguish the difference of those treatments has not yet been reported, thus we do not know which one is the best choice for future potential application. Here, we describe our systematic comparative analysis of silk fibroins prepared with four of the commonly used preparative solutions, Ca(NO_3_)_2_-methanol, Ca(NO_3_)_2_-ethanol, CaCl_2_-methanol, and CaCl_2_-ethanol. The results could help to reveal the mechanisms of properties of silk-derived matrix under different treating conditions and provide evidence to choose right solution to prepare silk fibroins for potential drug delivery applications.

## Materials and methods

### Materials

L-asparaginase (L-ASNase) from *E. coli* (10,000 IU) was purchased from Changzhou Qianhong Bio-Pherma Co., Ltd. (Jiangsu Province, China). L-asparagines’ (anhydrous) was purchased from Sangon Biotech (Shanghai) Co., Ltd. (Shanghai, China). Trichloroactic acid (TCA) was purchased from Sinopharm Chemical Reagent Co., Ltd. (Beijing, China). Methanol, ethanol, calcium nitrate tetrahydrate (Ca(NO_3_)_2_·4H_2_O), calcium chloride (CaCl_2_), and HgI_2_, all analytical reagent grade, were purchased from Chengdu Kelong Chemical Reagent Factory (Sichuan Province, China).

### Preparation of degummed silk fibroin

Cocoons from *B. mori* were degummed by incubating in a mixture of sodium dodecyl sulfate (SDS; 0.25%,w/v) and sodium carbonate (0.25%,w/v) at 98°C for 30 min. The samples were then cooled to room temperature, rinsed three times with deionized water, and dried at 65°C overnight. The ratio of cocoons and solution was 1:100 (w/v). The degummed silk fibroins were isolated, along with another silk protein, sericin.

### Calcium-alcohol solvents treatment of silk fibers

The isolated fibroin fibers were separately dissolved in concentrated CaCl_2_ solution mixed with ethanol or methanol and water (1:2:8 mol), and separately dissolved in concentrated Ca(NO_3_)_2_·4H_2_O solution mixed with ethanol or methanol (1:2 mol) at 65°C in a water bath for 1 h. The ratio of the silk fibers and solution was 1:20 (m/v). The aqueous solution of silk fibroin was obtained by dialyzing against flowing water. After that, the resulting dialyzed solutions were lyophilized. The dry silk powder (fibroins treated with CaCl_2_-ethanol solution) or pieces (from the other three solutions) were stored at 4°C until use.

### SEM

The silk fibroins were vacuum-coated with a 20 nm layer of gold. The surface morphology of each silk fibroin was observed with a scanning electron microscope (S-3400N SEM; Hitachi, Japan) and photographed at a voltage of 15 kV and room temperature.

### SDS-polyacrylamide gel electrophoresis (PAGE)

The silk fibroins separately treated with Ca(NO_3_)_2_-methanol, Ca(NO_3_)_2_-ethanol, CaCl_2_-methanol, and CaCl_2_-ethanol solution were analyzed by SDS-PAGE to determine the corresponding molecular weights of the protein. Samples were resolved on 12% acrylamide gel and 4% condensing gel, and protein bands were visualized by staining with 0.25% Coomassie Brilliant Blue R-250 (Sigma-Aldrich, St. Louis, MO, USA).

### FTIR spectroscopy

The infrared spectra of each fibroin produced with Ca(NO_3_)_2_-methanol, Ca(NO_3_)_2_-ethanol, CaCl_2_-methanol, and CaCl_2_-ethanol solution, and degummed fibroins (as control), were measured on a FTIR spectrometer using KBr pellets (Tensor 27 FTIR; Bruker, Ettlingen, Germany). Spectra, with a resolution of 4 cm^-1^, were recorded and subtracted from the sample readings. All samples were measured in reflection mode; for this, the silk fibroin powder treated with CaCl_2_-ethanol solution had been transformed into tablet form. The results are presented as the average of 64 repeated 4000 ~ 400 cm^-1^ scans.

### WAXD

The crystalline structure of the silk fibroins produced with Ca(NO_3_)_2_-methanol, Ca(NO_3_)_2_-ethanol, CaCl_2_-methanol, and CaCl_2_-ethanol solution, and of degummed fibroins, were determined by WAXD using a Siemens type F X-ray diffractometer (Siemens, Munich, Germany) with Ni-filtered Cu Kα radiation. The voltage and current of the X-ray source were 30KV and 20 mA, respectively. The wavelength, λ, was 0.15406 nm. The samples were mounted on aluminum frames and scanned from 5° to 40° (2θ) at a speed of 2°/min. The d-spacing was calculated by the following equation: D = λ/(2 × sin(θ)), i.e. D = 0.0752/(sin θ) nm. For example, if the scanning angel was 2θ = 20°, then D = 0.0752/(sin 10°) nm, and the D-space was 0.43 nm.

### Solid-state ^13^C CP/MAS-NMR spectra measurement

Solid state ^13^C CP/MAS-NMR has been successfully used to analyze the secondary structure of proteins [15], and was similarly applied in our study. The ^13^C CP/MAS-NMR spectra were recorded on a Bruker AVANCE III 400 WB spectrometer equipped with a 4 mm standard bore CP/MAS probe head, whose X channel was tuned to 100.62 MHz for ^13^C and the other channel was tuned to 400.18 MHz for broad band 1H decoupling. A magnetic field of 9.39T at 297 K was used. The dried and finely powdered samples were packed in a ZrO_2_ rotor that was sealed with an Kel-F cap and spun at 12 kHz rate. The experiments were conducted at a contact time of 2 ms. A total of 3000 scans were recorded with 6 s recycle delay for each sample. All ^13^C CP/MAS chemical shifts were referenced to the resonances of the adamantane (C10H16) standard (δCH2 = 38.5).

### Enzyme cross-linking and activity test

L-ASNase immobilization was performed according to the method previously described by Zhang et al. [16] with minor modifications. An aliquot (50 mg) of each fibroin produced with Ca(NO_3_)_2_-methanol, Ca(NO_3_)_2_-ethanol, CaCl_2_-methanol, and CaCl_2_-ethanol solution, and degummed fibroins (as control), were placed into plastic centrifuge tubes and mixed with 2mL L-ASNase in phosphate buffered saline (PBS) solution (2 mg/mL) and 1 mL L-asparagine PBS solution (5 mg/L). The L-asparagine acted as the enzyme activity center protector. After gentle shaking, glutaraldehyde (0.05%) and PBS (pH 7.4) were added to bring the final volume to 5 mL, and the solutions were incubated at 4°C overnight to facilitate the cross-linking reaction. The next day, the reaction was stopped by adding 100 mg glycine to each tube. The fibroin and L-ASNase bioconjugates were then washed with Tris–HCl buffer (pH 8.6) and purified by centrifugation in a J2-MI refrigerated centrifuge (Beckman-Coulter, Brea, CA, USA) at 10000 rpm for 10 min at 4°C; this purification process was repeated three times with intervening Tris–HCl washes. After the final spin, the pellets were each resuspended in 1 mL Tris–HCl and incubated in a water bath at 37°C for 10 min. Two milliliters of 5 mg/mL L-asparagine in Tris–HCl was added to each tube, and incubation continued at 37°C for another 10 min. The enzymatic reaction was terminated by adding 100 mg TCA to each reaction. The tubes were centrifuged at 3000 rpm for 5 min, and 0.5 mL of the supernatant from each was transferred to fresh centrifuge tubes containing 1 mL Nessler’s reagent. After the reaction processed at room temperature for a given time, 50 μL of each mixture and 150 μL of Tris–HCl buffer were transferred in triplicate to 96 well microplates. The same experiment was repeated three times. The activities of the enzymes attached to the fibroins were calculated by determining the change in optical density at 450 nm as measured on a microplate reader (Paradigm; Beckman-Coulter). Data are presented as mean ± SD and evaluated using the Student’s *t*-test (SPSS 13.0, SPSS Inc.). *P* < 0.05 was considered to be statistically significant. The mobilization and activity detection of glucose oxidase were performed by referring to the published literature [8].

## Results and discussion

### Morphology of silk fibroins

**Figure 1:**
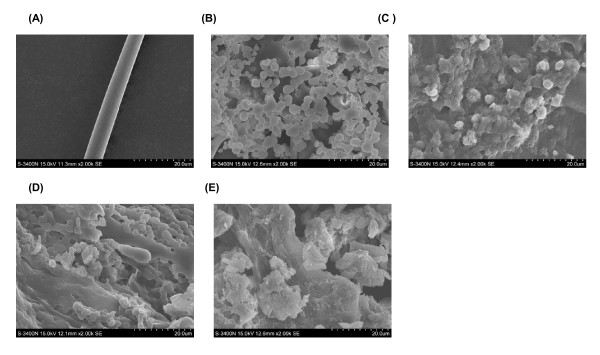
**SEM photographs of *B. mori *silk fibroin prepared with various solutions.** (**A**) Degummed silk fibroin. (**B**) Silk fibroin prepared from Ca(NO_3_)_2_·4H_2_O-methanol solution. (**C**) Silk fibroin prepared from Ca(NO_3_)_2_·4H_2_O-ethanol solution. (**D**) Silk fibroin prepared from CaCl_2_-methanol-H_2_O solution. (**E**) Silk fibroin prepared from CaCl_2_-ethanol-H_2_O solution.

The silk fibroins treated with Ca(NO_3_)_2_·4H_2_O-methanol, Ca(NO_3_)_2_·4H_2_O-ethanol, CaCl_2_-methanol-H_2_O, and CaCl_2_-ethanol solution were separately dissolved. After lyophilized, the surface morphology of degummed silk fibroins and regenerated silk fibroins was observed with SEM (Figure 1). The size and shape of the degummed silk fibroins were normal, with diameters of 6–8 μm (Figure 1A). In contrast, the regenerated silk fibroins were spherical or irregular shapes. This shape may have resulted from the merger of smaller micelles that occurred in the aqueous solutions of Ca(NO_3_)_2_·4H_2_O-methanol (Figure 1B), Ca(NO_3_)_2_·4H_2_O-ethanol (Figure 1C), and CaCl_2_-methanol-H_2_O (Figure 1D), and CaCl_2_-ethanol (Figure 1E). 

### Molecular weight ranges of silk fibroins

The silkworm’s cocoon is composed of two kinds of silk protein, the silk sericin, which makes up the membrane, and the silk fibroin, which makes up the inner portion. The silk sericin is a glue-like mixture of glycoproteins with varying molecular mass, and is removed by the degumming and rinsing steps. The silk fibroin protein of *B. mori* is rich in alanine, glycine and serine residues [17], and is ~400 kDa, with 300 kDa making up a heavy chain (H-chain), 26 kDa making up a light chain (L-chain), L-chain and H-chain linked by disulfide bond(s) and about 30 kDa making up a P25 glycoprotein that associates with the H-L complex primarily by hydrophobic interactions [18].

**Figure 2:**
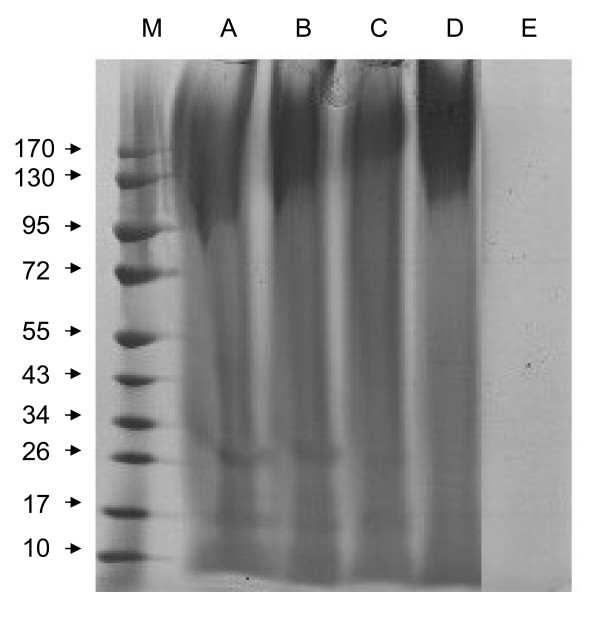
**SDS-PAGE analysis of *B. mori *silk fibroins prepared with various solutions.** Silk fibroins were prepared from four different calcium-alcohol solutions (as described below) then dissolved in hot water. The range of molecular weight of the proteins produced by each solution was determined by SDS-PAGE with 12% acrylamide gel and 4% condensing gel, which was stained with 0.25% Coomassie Brilliant Blue R-250. Lanes: M, marker. (**A**) silk fibroin prepared from Ca(NO_3_)_2_·4H_2_O-methanol solution. (**B**) silk fibroin prepared from Ca(NO_3_)_2_·4H_2_O-ethanol solution. (**C**) silk fibroin prepared from CaCl_2_-methanol-H_2_O solution. (**D**) silk fibroin prepared from CaCl_2_-ethanol-H_2_O solution. (**E**) Degummed silk fibroin.

The silk fibroins produced with Ca(NO_3_)_2_-methanol, Ca(NO_3_)_2_-ethanol, CaCl_2_-methanol, and CaCl_2_-ethanol solutions were dissolved, and the molecular weights were measured by SDS-PAGE. As shown in Figure 2, the regenerated silk fibroins treated with Ca(NO_3_)_2_-methanol had a molecular weight from about 95 KDa to over 170 kDa, but Ca(NO_3_)_2_-ethanol from about 100 KDa to over 170 kDa. The CaCl_2_-methanol solution fibroins ranged from about 140 to over 170 kDa, while the CaCl_2_-ethanol fibroins ranged from about 100 to nearly 300 kDa. Two low molecular weight bands, ~17 and ~26 kDa, were obviously present in these regenerated silk fibroins, but the silk fibroins produced with CaCl_2_-ethanol showed relatively faint low molecular weight bands at these positions. In addition, the degummed silk fibroins are poorly soluble, except in the chemistry solution and organic solvents, we could not observe obvious bands in the gel. 

This phenomenon suggested that some of the disulfide linkages and hydrophobic bonds, between silk fibroin molecules may have been destroyed by Ca(NO_3_)_2_-methanol, Ca(NO_3_)_2_-ethanol, or CaCl_2_-methanol treatments. The solvent of CaCl_2_-ethanol appeared to be sufficiently gentle to produce silk fibroins with less obvious damage to the secondary bonds. Thus, the CaCl_2_-ethanol solution may be superior to the other solutions in its ability to protect the integrity of the fibroin secondary structure. The regenerated silk protein treated with these calcium-alcohol solvents were water-soluble, this results consistent with published reports that the silk proteins prepared from Ca(NO_3_)_2_-methanol were water-soluble, indicating that the regenerating coagulants affected the crystallinity and conformation of the fibroin [14].

### Fourier-transform infrared spectroscopic analysis of the silk fibroins’ crystalline structure

Due to the presence of amide groups in silk protein, the characteristic vibration bands around 1620 cm^-1^ were assigned to the absorption peak of the peptide backbone of amide I (C = O stretching), bands around 1513 cm^-1^ to amide II (N-H bending), the bands around 1230 and 1444 cm^-1^ to amide III (C-N stretching) [15], and 694 cm^-1^ to amide IV [19,20]. All these characteristic absorbance peaks indicate the existence of a hydrogen-bonded NH group [21]. The molecular conformation of *B. mori* silk fibroin is characterized by β-sheet absorption peaks around 1630, 1530 and 1240 cm^−1^, random coil conformation absorption peaks at 1650 or 1645, 1550 and 1230 cm^−1^, and an α-helix absorption peak around 1655 cm^−1^[15,22]. Tang and colleagues had previously reported that the intensity of peaks around 3300 cm^-1^ (data not shown here) fluctuate in response to hydrogen bonds [23].

**Figure 3:**
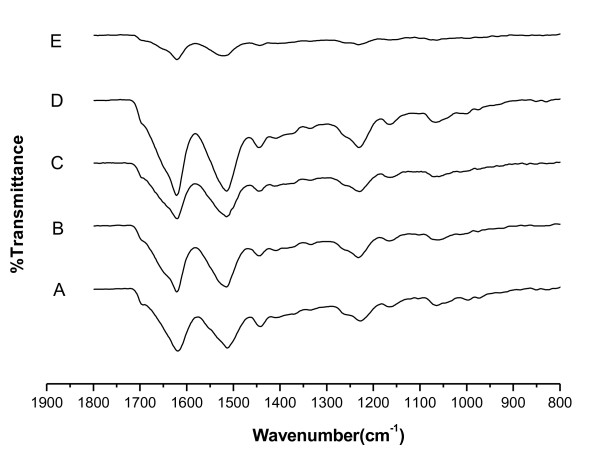
**FTIR transmittance spectra of *B. mori *silk fibroins prepared with various solutions.** (**A**) Degummed silk fibroin. (**B**) Silk fibroin prepared from Ca(NO_3_)_2_·4H_2_O-methanol solution. (**C**) Silk fibroin prepared from Ca(NO_3_)_2_·4H_2_O-ethanol solution. (**D**) Silk fibroin prepared from CaCl_2_-methanol-H_2_O solution. (**E**) Silk fibroin prepared from CaCl_2_-ethanol-H_2_O solution.

In Figure 3, the β-sheet conformation was indicated by shifts of absorption peaks as follows: 1625-1630 cm^−1^ (amide I), 1520-1530 cm^−1^ (amide II), and 1265-1270 cm^−1^ (amide III). FTIR spectra of the regenerated silk fibroins showed intense absorption peaks around 1620 cm^-1^, 1514 cm^-1^, and 1230 cm^-1^, which are the characteristic absorption peaks of β-sheet. The detected crystalline structure of CaCl_2_-ethanol silk fibroin showed more silk I (α-form, type II β-turn), while that of the other three fibroins showed more silk II (β-form, anti-parallel β-pleated sheet). 

### Wide-angle X-ray diffraction analysis of the silk fibroins’ crystalline structure

The toughness of silk fibers is dependent on their β-sheet composition. In spider and cocoon silk, the β-sheet consists of a poly-alanine or a GAGAGAGAAS sequence, arranged in an anti-parallel or parallel conformation. A previous study by Lu *et al.* demonstrated that the corresponding D-spacings of silk I (α-form, type II β-turn) were 0.74 nm, 0.56 nm, 0.44 nm, 0.41 nm, 0.36 nm, 0.32 nm, and 0.28 nm, and of silk II (β-form, anti parallel β-pleated sheet) were 0.98 nm, 0.48 nm, and 0.43 nm [24].

**Figure 4:**
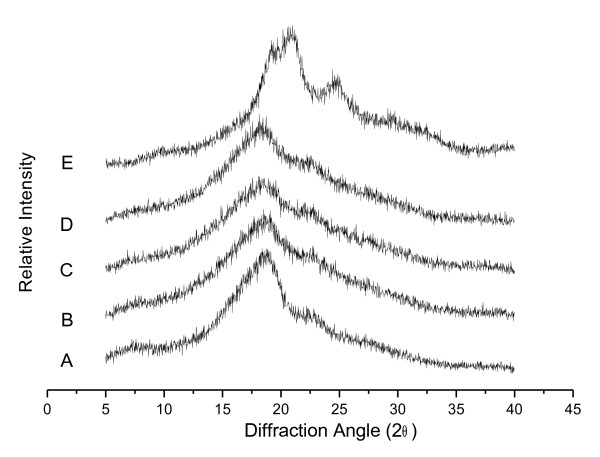
**Wide-angle X-ray diffraction patterns of *B. mori *silk fibroins prepared with various solutions.** (**A**) Degummed silk fibroin. (**B**) Silk fibroin prepared from Ca(NO_3_)_2_·4H_2_O-methanol solution. (**C**) Silk fibroin prepared from Ca(NO_3_)_2_·4H_2_O-ethanol solution. (**D**) Silk fibroin prepared from CaCl_2_-methanol-H_2_O solution. (**E**) Silk fibroin prepared from CaCl_2_-ethanol-H_2_O solution.

Figure 4 shows the WAXD data of the regenerated silk fibroins produced in our study with Ca(NO_3_)_2_-methanol, Ca(NO_3_)_2_-ethanol, CaCl_2_-methanol, and CaCl_2_-ethanol solutions. The degummed fibroin and fibroins produced with Ca(NO_3_)_2_-methanol, Ca(NO_3_)_2_-ethanol, and CaCl_2_-methanol showed similar 2θ diffraction peaks, which corresponded to silk II crystalline spacing of 0.47 nm (2θ = 18.4°), and silk I crystalline spacing of 0.39 nm (2θ = 22.4^o^). In contrast, fibroin treated with CaCl_2_-ethanol showed four obvious diffraction peaks at 2θ, namely 19.4°, 20.3°, 24.6°, and 29.3°, which corresponded to silk I crystalline spacing of 0.44 nm, 0.41 nm, 0.35 nm, and 0.30 nm, respectively. No typical diffraction peaks of silk II were found for this regenerated silk fibroin. The mean peak at 2θ = 18.4° for fibroins treated with Ca(NO_3_)_2_-methanol, Ca(NO_3_)_2_-ethanol, or CaCl_2_-methanol was not as sharp as that for degummed fibroin. This finding indicated that these solutions decreased the crystallization ability of fibroin, and the sharp peak observed at 2θ = 20.3° of CaCl_2_-ethanol fibroins indicated an increased crystallization ability of fibroin. 

Furthermore, the fibroins produced with CaCl_2_-ethanol were composed of more silk I (α-form, type II β-turn) than the other three regenerated fibroins and the degummed fibroin, which had more silk II (β-form, anti-parallel β-pleated sheet). As reported previously, the standard orientation methods, such as rolling and drawing, are able to transform the metastable silk I into silk II [25]. Therefore, it is possible that the CaCl_2_-ethanol solution is superior to the other solutions in its ability to protect the integrity of the fibroin, maintaining more silk I.

### Solid-state ^13^C CP/MAS-NMR analysis of the conformational and inter-molecular arragement of silk fibroins

**Figure 5:**
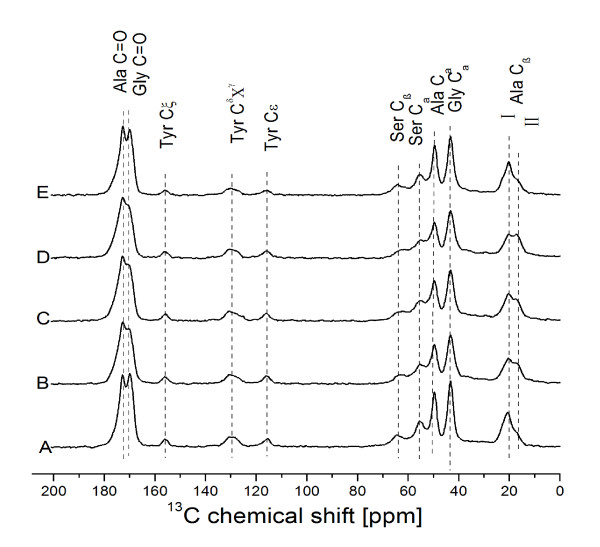
**Solid-state**^**13**^**C CP/MAS-NMR spectra of *B. mori *silk fibroins prepared with various solutions.** (**A**) Degummed silk fibroin. (**B**) Silk fibroin prepared from Ca(NO_3_)_2_·4H_2_O-methanol solution. (**C**) Silk fibroin prepared from Ca(NO_3_)_2_·4H_2_O-ethanol solution. (**D**) Silk fibroin prepared from CaCl_2_-methanol-H_2_O solution. (**E**) Silk fibroin prepared from CaCl_2_-ethanol-H_2_O solution.

As shown in Figure 5, the peaks on the dotted line marked Ala C_β_ II (16.8 ppm) correspond to the representative peaks of random coil or distorted β-turn, where the torsion angles of a backbone chain are distributed largely around the averaged angles of silk II structure [26]. The other two peaks that were observed, at 20.2 and 22.6 ppm, correspond to anti-parallel β-sheets [27]. However, the presence of peaks of Ala C_β_ at 15.2 ppm and Ala C_α_ at 52.4 ppm suggested that the residues strongly favor an ordered structure, most likely a helical structure [28]. According to previous studies, the silk protein consists of many repeated motif sequences, such as AGSGAG [29], AGYGAG, AGVGYGAG and GAAS [30]; since glycine and alanine can readily form peptide bonds, this is a likely event in the regenerated silk fibroins. 

The peaks of alanine and serine carbons that were observed in our study samples suggest that all of them contain random coils or distorted β-turns (16.8 ppm peak). An increased amount of these peaks was found in the fibroins produced with Ca(NO_3_)_2_-methanol, Ca(NO_3_)_2_-ethanol, and CaCl_2_-methanol, as compared with degummed silk fibroins and silk fibroins treated with CaCl_2_-ethanol (Figure 5).

The peak position of the C_α_ and C_β_ carbons from alanine and serine residues indicate clearly that these samples had a β-sheet structure. The silk fibroins produced with Ca(NO_3_)_2_-methanol, Ca(NO_3_)_2_-ethanol, and CaCl_2_-methanol solutions showed increased peaks for alanine C_β_ and decreased peaks for glycine with C = O (169.1 ppm peak) interactions between them. Furthermore, the peaks of silk fibroin produced with CaCl_2_-ethanol were nearly identical to those for the degummed silk fibroin sample. This finding may be related to the different levels of chemical shift that were produced by the Ca(NO_3_)_2_-methanol, Ca(NO_3_)_2_-ethanol, and CaCl_2_-methanol solutions. Regardless, the regenerated fibroin produced with CaCl_2_-ethanol solution appeared to be the best method to protect the conformational and inter-molecular arrangement of the silk fibroin chains of degummed fibroin.

### Enzymatic activity of L-ASNase when conjugated with silk fibroins

L-asparaginase is a well-established chemotherapeutic agent in routine use to treat acute lymphoblastic leukemia. However, treatment withdrawal due to side effects, some life-threatening and immunological reactions is not uncommon [31]. In addition, circulation half-life is short, necessitating longer and larger doses of the drug.

**Figure 6:**
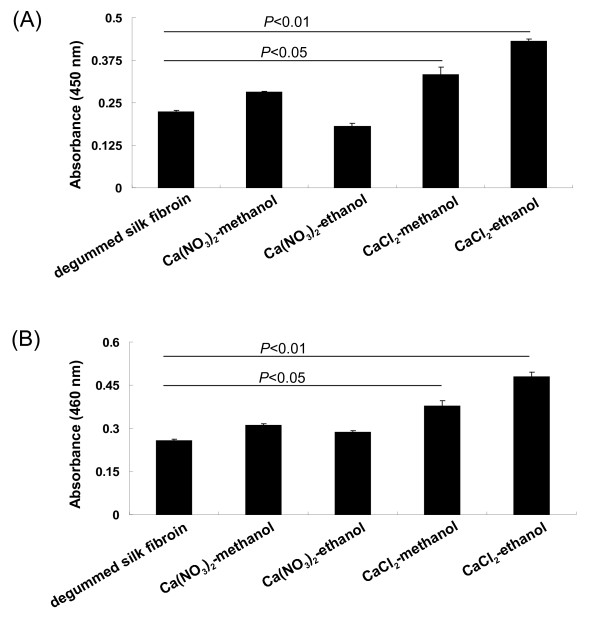
**Enzyme activity tests for silk fibroins prepared with various solutions.** L-ASNase (**A**) and glucose oxidase (**B**) were separately immobilized to degummed silk fibroin or regenerated fibroins prepared from four different calcium-alcohol solutions. The activities of these enzymes attached to the fibroins were calculated as a change in optical density at 450 or 460 nm measured on a microplate reader, accordingly. Results are presented as mean ± SD (n = 3 assays of triplicate samples). *P* < 0.05 was considered to be statistically significant.

In this study, we tested whether any of the four silk fibroins produced by the different solutions had more beneficial effects on a bioconjugated enzyme that is relevant for human therapy. The L-ASNase enzyme was chosen for these *in vitro* experiments, along with its substrate L-asparagine. Since L-ASNase hydrolysis of L-asparagine produces NH_3_, Nessler’s reagent, which turns yellow in the presence of NH_3_, was chosen to measure L-ASNase activity. According to the results, the activity of L-ASNase attached to the regenerated fibroins produced with CaCl_2_-ethanol solution was higher than the other fibroins (Figure 6A). The highest activity of glucose oxidase-linked to the four silk fibroins was observed in CaCl_2_-ethanol group too, very similar to that observed for L-ASNase (Figure 6B). Therefore, the CaCl_2_-ethanol solution appear to be the most appropriate methods by which to prepare regenerated silk fibroins for use as drug delivery carriers, at least for these two particular enzymes. However, the immunogenicity and biocompatibility properties of these regenerated silk fibroins produced with CaCl_2_-ethanol have yet to be determined and require further investigation in an animal model before clinical application. 

Silk is a unique protein biopolymer, with a block copolymer structure dominated by large hydrophobic domains and small hydrophilic spacers. This primary structure, upon folding into assembled silk structures, leads to organized crystalline domains (β-sheets) and less organized more flexible domains (more hydrated). This assembly leads to localized nanoscale pockets where other proteins may be entrapped with limited but sufficient hydration [8]. The silk biomaterial offers some important features that suggest utility as a stabilization matrix. In addition, methanol and ethanol treatment of silk fibroin resulted in a gradual transition from silk I to silk II [32,33]. The analyses also indicated that formation of aggregated strands among extended sericin chains induced by ethanol treatment is the key to generating molecular orientation [34]. Other research showed that the chimeric protein which formed by a clone encoding consensus repeats from the major protein in the spider dragline silk of Nephila clavipes fused to the carboxyl terminal domain of dentin matrix protein 1 (CDMP1) was incubated with CaCl_2_, the secondary structure shifted from random coil to α-helix and β-sheet, due to the interactions between the CDMP1 domain and Ca^2+^[35]. The results confirmed that concentrated neutral salts such as Ca^2+^, or organic solvents including methanol and ethanol can affected the crystallinity and conformation of the fibroin. However, the molecular mechanisms for these effects have to be clarified in the future.

The degradation rate of a matrix is an important parameter for a biomaterial designed to be used for tissue engineering applications. The properties of silk-matrix also directly affect the enzymatic degradation of the enzymes attached. Random coil and α-helical structures formed of the biospun fibroin accelerate the process of degradation in both PBS and enzyme solutions in comparison with β-sheets [36]. Zhang *et al.* reported that cross-linking L-ASNase with regenerated silk fibroin treated with CaCl_2_-ethanol solution significantly increased heat and storage stability and resistance to trypsin digestion, and its longer half-life (63 h) than that of control L-ASNase (33 h) [9]. These observations also suggested that the silk-based matrix prepared with CaCl_2_-ethanol solution formed more crystalline domains (β-sheets) potentially help to decrease the degradation rate.

It’s concluded that regenerated silk fibroin can be used as an immobilization matrix for enzymes or therapeutic proteins. The properties of the regenerated silk-matrix directly effect the activity and stability of the enzymes attached. In the present research, Fourier-transform infrared spectroscopy and X-ray diffraction showed that the regenerated silk-matrix treated with CaCl_2_-ethanol has a crystalline structure with more silk I (α-form, type II β-turn), while the silk-matrixes treated with other solutions have more silk II (β-form, anti-parallel β-pleated sheet). Solid-State ^13^C CP/MAS-NMR analysis also suggested that the silk-matrix regenerated from CaCl_2_-ethanol were nearly identical to degummed silk fibroin, while the others show significantly different chemical shifts. These results suggested that the silk-based matrix prepared with CaCl_2_-ethanol solution formed more crystalline domains (β-sheets) than others, which potentially helps to enhance the stability and improve activity of drug or therapeutic proteins. Furthermore, the properties of the regenerated silk-matrix can satisfy the needs of modern carrier materials, ruling out the use of most synthetic polymer materials, thus the carrier materials of silk fibroin treated with CaCl_2_-ethanol could be widely applicable.

In addition, a range of medical needs such as silk sutures, drug delivery systems, and fiber-based tissue products that exploit the mechanical properties of silks can be envisioned for ligament, bone, and other tissue repairs may become more and more popular in the next few years [1]. These materials based on silk fiber can lead to multifunctional material platforms that integrate with living systems for medical materials, industrial material and a host of other applications.

## Conclusions

Preparation of *B. mori* degummed silk fibroin by CaCl_2_-ethanol preserved the best original protein structure and produced a better affinity to the enzyme drug L-ASNase than the Ca(NO_3_)_2_-methanol, Ca(NO_3_)_2_-ethanol and CaCl_2_-methanol treatments. The CaCl_2_-ethanol solution may represent the most appropriate method by which to prepare silk fibroins for use as biomaterials, especially as carriers for drug delivery.

## Competing interests

The authors declare that they have no competing interests.

## Authors’ contributions

Hao Zhang and Ling-ling Li mainly performed research; in addition, Hao Zhang analyzed data and wrote the paper. Xia Yang designed research, analyzed data and wrote the paper. Fang-yin Dai, Hao-hao Zhang and Wei Zhou helped to prepare the materials and performed research. Bing Ni revised the manuscript. Yu-zhang Wu participated in the design of the study and provided administrative support. All authors have contributed and approved the final manuscript.

## References

[B1] Omenetto FG Kaplan DL New opportunities for an ancient material Science 2010 329 528 531 20671180 10.1126/science.1188936 PMC3136811

[B2] Lovett M Eng G Kluge JA Cannizzaro C Vunjak-Novakovic G Kaplan DL Tubular silk scaffolds for small diameter vascular grafts Organogenesis 2010 6 217 224 21220960 10.4161/org.6.4.13407 PMC3055647

[B3] Altman GH Diaz F Jakuba C Calabro T Horan RL Chen J Lu H Richmond J Kaplan DL Silk-based biomaterials Biomaterials 2003 24 401 416 12423595 10.1016/s0142-9612(02)00353-8

[B4] Leal-Egana A Scheibel T Silk-based materials for biomedical applications Biotechnol Appl Biochem 2010 55 155 167 20222871 10.1042/BA20090229

[B5] Mauney JR Cannon GM Lovett ML Gong EM Di Vizio D Gomez P 3rd Kaplan DL Adam RM Estrada CR Jr Evaluation of gel spun silk-based biomaterials in a murine model of bladder augmentation Biomaterials 2011 32 808 818 20951426 10.1016/j.biomaterials.2010.09.051 PMC3742077

[B6] Ali MM Arumugam SB Effect of crude extract of Bombyx mori coccoons in hyperlipidemia and atherosclerosis J Ayurveda Integr Med 2011 2 72 78 21760692 10.4103/0975-9476.82527 PMC3131775

[B7] Zhang YQ Ma Y Xia YY Shen WD Mao JP Zha XM Shirai K Kiguchi K Synthesis of silk fibroin-insulin bioconjugates and their characterization and activities in vivo J Biomed Mater Res B Appl Biomater 2006 79 275 283 16767720 10.1002/jbm.b.30539

[B8] Lu S Wang X Lu Q Hu X Uppal N Omenetto FG Kaplan DL Stabilization of enzymes in silk films Biomacromolecules 2009 10 1032 1042 19323497 10.1021/bm800956n PMC2705330

[B9] Zhang YQ Zhou WL Shen WD Chen YH Zha XM Shirai K Kiguchi K Synthesis, characterization and immunogenicity of silk fibroin-L-asparaginase bioconjugates J Biotechnol 2005 120 315 326 16102867 10.1016/j.jbiotec.2005.06.027

[B10] Chatterjee S Barbora L Cameotra SS Mahanta P Goswami P Silk-fiber immobilized lipase-catalyzed hydrolysis of emulsified sunflower oil Appl Biochem Biotechnol 2009 157 593 600 19002611 10.1007/s12010-008-8405-y

[B11] Inoue S Matsunaga Y Iwane H Sotomura M Nose T Entrapment of phenylalanine ammonia-lyase in silk fibroin for protection from proteolytic attack Biochem Biophys Res Commun 1986 141 165 170 3800993 10.1016/s0006-291x(86)80349-7

[B12] Greish K Frandsen J Scharff S Gustafson J Cappello J Li D O'Malley BW Jr Ghandehari H Silk-elastinlike protein polymers improve the efficacy of adenovirus thymidine kinase enzyme prodrug therapy of head and neck tumors J Gene Med 2010 12 572 579 20603862 10.1002/jgm.1469 PMC2906606

[B13] Zhu ZH Ohgo K Asakura T Preparation and characterization of regenerated Bombyx mori silk fibroin fiber with high strength Express Polymer Letters 2008 2 885 889

[B14] Ha SW Park YH Hudson SM Dissolution of Bombyx mori silk fibroin in the calcium nitrate tetrahydrate-methanol system and aspects of wet spinning of fibroin solution Biomacromolecules 2003 4 488 496 12741761 10.1021/bm0255948

[B15] Ha SW Tonelli AE Hudson SM Structural studies of Bombyx mori silk fibroin during regeneration from solutions and wet fiber spinning Biomacromolecules 2005 6 1722 1731 15877399 10.1021/bm050010y

[B16] Zhang YQ Tao ML Shen WD Zhou YZ Ding Y Ma Y Zhou WL Immobilization of L-asparaginase on the microparticles of the natural silk sericin protein and its characters Biomaterials 2004 25 3751 3759 15020151 10.1016/j.biomaterials.2003.10.019

[B17] Lotz B Colonna Cesari F The chemical structure and the crystalline structures of Bombyx mori silk fibroin Biochimie 1979 61 205 214 465571 10.1016/s0300-9084(79)80067-x

[B18] Tanaka K Inoue S Mizuno S Hydrophobic interaction of P25, containing Asn-linked oligosaccharide chains, with the H-L complex of silk fibroin produced by Bombyx mori Insect Biochem Mol Biol 1999 29 269 276 10319440 10.1016/s0965-1748(98)00135-0

[B19] Chuan Xin Liang KH Improvements of the physical properties of fibroin membranes with sodium alginate J Appl Polym Sci 1992 45 1937 1943

[B20] Anshu Bagga Mathur AT Thomas Rathke Sam Hudson The dissolution and characterization of Bombyx mori silk fibroin in calcium nitrate-methanol solution and the regeneration of films Biopolymers 1997 42 61 74

[B21] Freddi G Pessina G Tsukada M Swelling and dissolution of silk fibroin (Bombyx mori) in N-methyl morpholine N-oxide Int J Biol Macromol 1999 24 251 263 10342772 10.1016/s0141-8130(98)00087-7

[B22] Meinel L Hofmann S Karageorgiou V Kirker-Head C McCool J Gronowicz G Zichner L Langer R Vunjak-Novakovic G Kaplan DL The inflammatory responses to silk films in vitro and in vivo Biomaterials 2005 26 147 155 15207461 10.1016/j.biomaterials.2004.02.047

[B23] Tang Y Cao C Ma X Chen C Zhu H Study on the preparation of collagen-modified silk fibroin films and their properties Biomed Mater 2006 1 242 246 18458412 10.1088/1748-6041/1/4/010

[B24] Lu Q Hu X Wang X Kluge JA Lu S Cebe P Kaplan DL Water-insoluble silk films with silk I structure Acta Biomater 2010 6 1380 1387 19874919 10.1016/j.actbio.2009.10.041 PMC2830340

[B25] He SJ Valluzzi R Gido SP Silk I structure in Bombyx mori silk foams Int J Biol Macromol 1999 24 187 195 10342764 10.1016/s0141-8130(99)00004-5

[B26] Zhu Z Kikuchi Y Kojima K Tamura T Kuwabara N Nakamura T Asakura T Mechanical properties of regenerated Bombyx mori silk fibers and recombinant silk fibers produced by transgenic silkworms J Biomater Sci Polym Ed 2010 21 395 411 20178693 10.1163/156856209X423126

[B27] Yao J Asakura T Synthesis and structural characterization of silk-like materials incorporated with an elastic motif J Biochem 2003 133 147 154 12761210 10.1093/jb/mvg014

[B28] Zhao C Yao J Masuda H Kishore R Asakura T Structural characterization and artificial fiber formation of Bombyx mori silk fibroin in hexafluoro-iso-propanol solvent system Biopolymers 2003 69 253 259 12767126 10.1002/bip.10350

[B29] Fraser RD MacRae TP Stewart FH Poly-l-alanylglycyl-l-alanylglycyl-l-serylglycine: a model for the crystalline regions of silk fibroin J Mol Biol 1966 19 580 582 5969080 10.1016/s0022-2836(66)80026-8

[B30] Laemmli UK Cleavage of structural proteins during the assembly of the head of bacteriophage T4 Nature 1970 227 680 685 5432063 10.1038/227680a0

[B31] Killander D Dohlwitz A Engstedt L Franzen S Gahrton G Gullbring B Holm G Holmgren A Hoglund S Killander A Hypersensitive reactions and antibody formation during L-asparaginase treatment of children and adults with acute leukemia Cancer 1976 37 220 228 1061636 10.1002/1097-0142(197601)37:1<220::aid-cncr2820370132>3.0.co;2-w

[B32] Wilson D Valluzzi R Kaplan D Conformational transitions in model silk peptides Biophys J 2000 78 2690 2701 10777765 10.1016/S0006-3495(00)76813-5 PMC1300858

[B33] Zhang K Fan L Yan Z Yu Q Mo X Electrospun Biomimic Nanofibrous Scaffolds of Silk Fibroin/Hyaluronic Acid for Tissue Engineering J Biomater Sci Polym Ed 2011 22 1069 1082 21722417 10.1163/092050611X576963

[B34] Teramoto H Miyazawa M Molecular orientation behavior of silk sericin film as revealed by ATR infrared spectroscopy Biomacromolecules 2005 6 2049 2057 16004444 10.1021/bm0500547

[B35] Huang J Wong C George A Kaplan DL The effect of genetically engineered spider silk-dentin matrix protein 1 chimeric protein on hydroxyapatite nucleation Biomaterials 2007 28 2358 2367 17289141 10.1016/j.biomaterials.2006.11.021

[B36] Mandal BB Kundu SC Biospinning by silkworms: silk fiber matrices for tissue engineering applications Acta Biomater 2010 6 360 371 19716447 10.1016/j.actbio.2009.08.035

